# Nucleotide Excision Repair: Insights into Canonical and Emerging Functions of the Transcription/DNA Repair Factor TFIIH

**DOI:** 10.3390/genes16020231

**Published:** 2025-02-19

**Authors:** Amélie Zachayus, Jules Loup-Forest, Vincent Cura, Arnaud Poterszman

**Affiliations:** 1 Institut de Génétique et de Biologie Moléculaire et Cellulaire (IGBMC), 1 Rue Laurent Fries, 67400 Illkirch-Graffenstaden, France; amelie.zachayus@igbmc.fr (A.Z.); jules.loup-forest@igbmc.fr (J.L.-F.); vincent.cura@igbmc.fr (V.C.); 2 Centre National de la Recherche Scientifique (CNRS), UMR 7104, 1 Rue Laurent Fries, 67400 Illkirch-Graffenstaden, France; 3 Institut National De La Sante et de la Recherche Médicale (Inserm), UMR S 1258, 1 Rue Laurent Fries, 67400 Illkirch-Graffenstaden, France; 4 Equipe Labellisée Ligue Contre le Cancer, Institut de Génétique et de Biologie Moléculaire et Cellulaire (IGBMC), 1 Rue Laurent Fries, 67400 Illkirch-Graffenstaden, France

**Keywords:** DNA repair, transcription, NER, TFIIH

## Abstract

Nucleotide excision repair (NER) is a universal cut-and-paste DNA repair mechanism that corrects bulky DNA lesions such as those caused by UV radiation, environmental mutagens, and some chemotherapy drugs. In this review, we focus on the human transcription/DNA repair factor TFIIH, a key player of the NER pathway in eukaryotes. This 10-subunit multiprotein complex notably verifies the presence of a lesion and opens the DNA around the damage via its XPB and XPD subunits, two proteins identified in patients suffering from Xeroderma Pigmentosum syndrome. Isolated as a class II gene transcription factor in the late 1980s, TFIIH is a prototypic molecular machine that plays an essential role in both DNA repair and transcription initiation and harbors a DNA helicase, a DNA translocase, and kinase activity. More recently, TFIIH subunits have been identified as participating in other cellular processes, including chromosome segregation during mitosis, maintenance of mitochondrial DNA integrity, and telomere replication.

## 1. Introduction

Genomic DNA is continuously subjected to damage from both endogenous and environmental genotoxic agents. These agents, which can originate from physical and chemical sources, include X-rays, ultraviolet (UV) radiation, and a wide array of chemicals [1,2,3]. If unrepaired, DNA damage can lead to persistent lesions that either trigger cell death by apoptosis or cause mutations, potentially resulting in cancer. Genomic instability is also linked to aging and a range of genetic diseases, highlighting the importance of cells being able to detect and repair damage to maintain cellular integrity and overall health [4,5]. To cope with the diversity of DNA threats, cells have developed complex mechanisms, collectively known as the DNA damage response, which detects DNA lesions, delays genome replication, opens chromatin, and either repairs the damage or leads the cells to senescence or apoptosis [6,7,8,9].

Among the various DNA repair mechanisms, nucleotide excision repair (NER) identifies and removes DNA-distorting lesions, including UV photoproducts like cyclobutane pyrimidine dimers (CPDs) or (6–4) pyrimidine–pyrimidone (6-4PPs), as well as DNA adducts from environmental mutagens such as polycyclic aromatic hydrocarbons found in tobacco smoke, and intrastrand cross-links caused by chemotherapeutic agents [10,11].

This review focuses on the human transcription/DNA repair factor TFIIH. It covers key discoveries related to the NER pathway and TFIIH and current insights into the structure and function of its 10-subunit complex in DNA repair, transcription, and non-canonical functions. This review also explores the molecular basis of TFIIH-associated diseases and its potential as a drug target.

## 2. The History of NER and the Discovery of TFIIH

**Figure 1:**
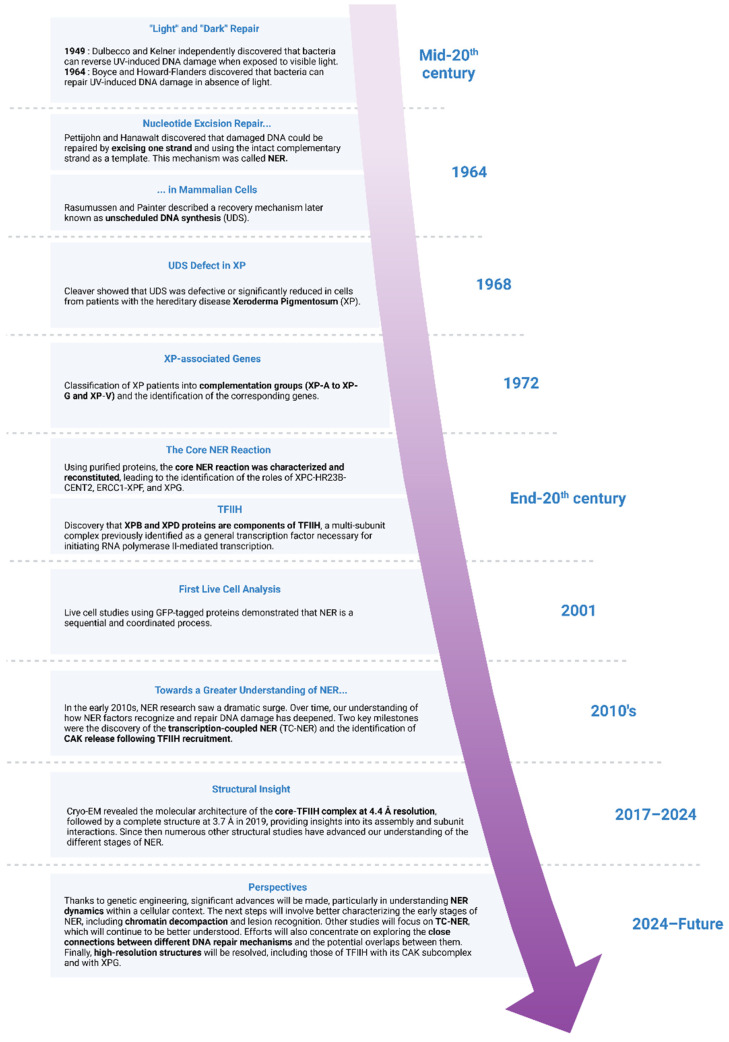
History of NER and discovery of TFIIH. NER was first identified in the mid-20th century, and significant advancements were made in the following decades, including the identification of Xeroderma Pigmentosum disorders and the discovery of the TFIIH complex by the end of the century. Since then, extensive research has deepened our understanding of TFIIH’s critical role in NER. However, further investigations are still required to fully unravel the complexities of TFIIH’s function, not only in NER but also in other cellular processes.

The history of NER dates back to pioneering findings in the mid-20th century when Renato Dulbecco and Albert Kelner independently discovered that bacteria could reverse UV-induced DNA damage when exposed to visible light, a process termed photoreactivation [12,13]. Subsequent research revealed that cells could repair UV-induced DNA damage even in the absence of light, indicating the existence of light-independent DNA repair mechanisms. These findings led to the concept of “dark repair”, contrasting with light-dependent repair [14,15,16] (Figure 1). Simultaneously, experiments based on density labeling (a technique used to examine DNA replication) showed that one strand of damaged DNA could be removed, with the resulting gap repaired by using the intact complementary strand as a template [17]. This process was termed nucleotide excision repair or “NER” because it involves the excision of a short DNA segment containing the damage. In the same year, Ronald Rasmussen and Robert Painter provided evidence for the repair of UV-damaged DNA in cultured mammalian cells, describing a recovery mechanism later known as repair synthesis, repair replication, or unscheduled DNA synthesis (UDS) [18]. A few years later, James Cleaver showed that UDS was defective or significantly reduced in cells from patients with the hereditary disease Xeroderma Pigmentosum (XP). Since patients with XP develop skin cancers upon exposure to sunlight, it has been suggested that the inability to repair DNA in their skin cells is linked to carcinogenesis, with certain human genes involved in NER being particularly implicated [19].

The understanding of NER in eukaryotes was advanced by foundational studies in yeast and humans, which not only elucidated the molecular characteristics of the pathway but also highlighted its evolutionary conservation across species. Our knowledge of NER in humans greatly benefited from complementation experiments, in which cells from different XP patients were fused and assessed for UDS [20]. This approach led to the classification of XP into eight complementation groups, i.e., XP-A to -G, where each group corresponds to causative mutations in one of the protein-coding genes involved in NER, as well as a variant form XP-V affecting a translesion DNA polymerase [21,22].

Advances in the NER field were then facilitated by the development of cell-free systems reconstituted from patient cell extracts [23]. These studies established that the human nucleotide excision nuclease removes thymine dimers from DNA by incising the 22nd phosphodiester bond 5′ and the 6th phosphodiester bond 3′ to the photodimer [24]. Further studies involving proteins purified from human cells or derived from recombinant sources led to the biochemical characterization of the core NER factors including Xeroderma Pigmentosum group C protein (XPC), which works in conjunction with homologous Rad23B (HR23B) and centrin-2 (CENT2) [25,26], as well as the endonucleases Excision Repair Cross-Complementing 1–Xeroderma Pigmentosum group protein F (ERCC1-XPF) [27,28] and Xeroderma Pigmentosum group G protein (XPG) [29], responsible for the 3′ and 5′ incisions, respectively.

A significant breakthrough came with the discovery that Xeroderma Pigmentosum group B protein (XPB) and Xeroderma Pigmentosum group D protein (XPD) are components of the transcription factor II H (TFIIH), a multi-subunit complex previously identified as a general transcription factor necessary for initiating RNA polymerase II-mediated transcription. Indeed, TFIIH was isolated almost simultaneously from rat liver (where it was named factor δ), yeast (factor b), and human Hela cells (BTF2 and later renamed TFIIH) for its ability to initiate transcription at RNA polymerase II (RNA Pol II) promoters using in vitro run-off assays [30,31,32,33]. In addition to its previously characterized carboxyl-terminal domain kinase activity [34], strand displacement assays established that the highly purified preparation of TFIIH (BTF2) exhibits ATP-dependent DNA helicase activities with opposite polarities [35,36]. Amino acid sequence analysis of the tryptic digest from the 89- and 80-kilodalton subunits of TFIIH revealed that these polypeptides corresponded to the XPB (ERCC-3) and XPD (ERCC-2) gene products, respectively, which are two presumed helicases implicated in the human DNA excision repair disorders XP and Cockayne syndrome (CS). These findings unveiled an unexpected direct molecular link between transcription and DNA repair, further supported by the identification of Rad25 (also known as Ssl2, the XPB yeast homolog) and Rad3 (the XPD yeast homolog) as subunits of TFIIH in yeast [37]. Experiments showing that purified TFIIH can rescue NER deficiencies of mammalian cell lines or yeast strains with mutations in the XPB or the XPD genes definitively established that TFIIH is a bona fide NER factor [35,36,38,39,40]. These in vitro experiments thus enabled the definition of the minimal set of factors necessary for the NER process [41,42,43,44]. Later, they were complemented by the analysis of protein dynamics in living cells using proteins tagged with the green fluorescent protein (GFP) and quantitative live cell microscopy [45,46]. These studies provided new insights into NER, particularly that NER functions through the sequential and coordinated assembly of the factors involved at the sites of UV damage.

## 3. The NER Pathways

### 3.1. From DNA Damage Detection to the Recruitment of TFIIH

**Figure 2:**
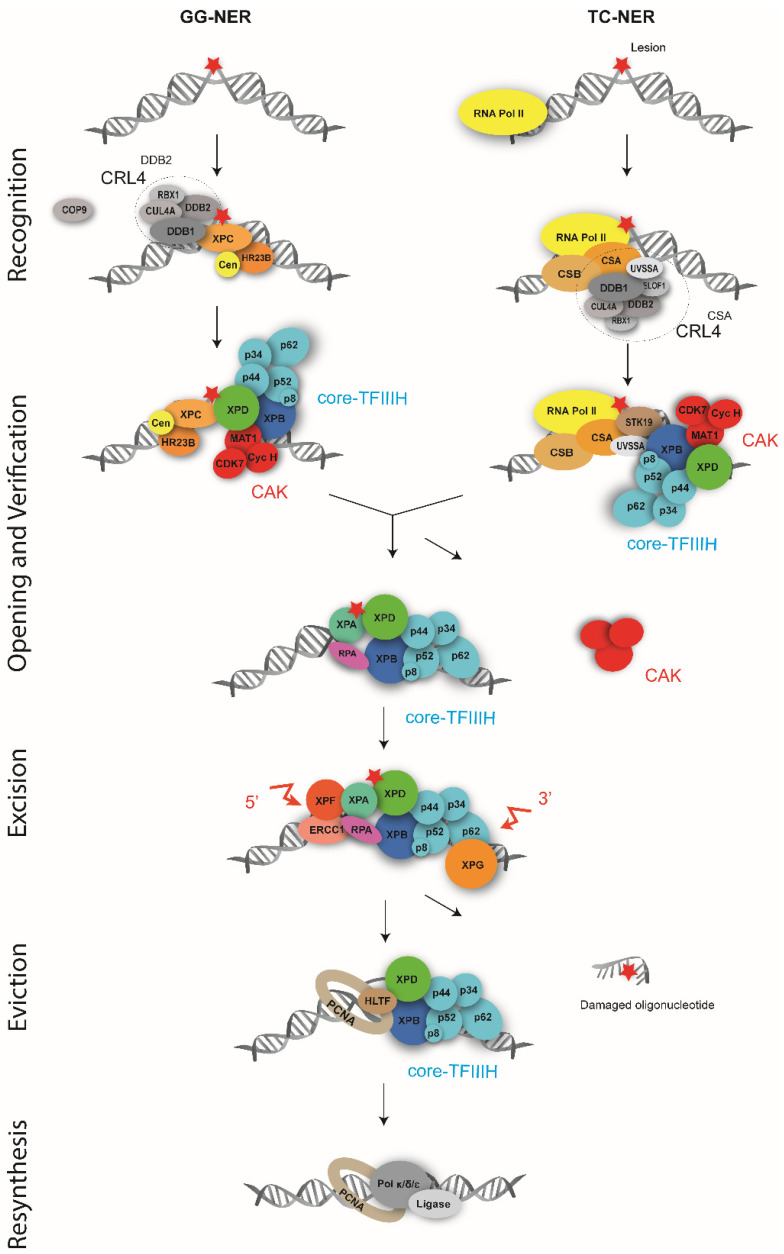
Schematic representation of NER. DNA damages are recognized in different ways depending on whether the DNA is transcriptionally active (via transcription-coupled repair, TC-NER) or inactive (via global genome repair, GG-NER). In GG-NER, DNA-distorting lesions are recognized by XPC in complex with Centrin 2 and HR23B. Lesions that cause minor distortion of the DNA double helix, such as CPDs, are detected by the CRL4^DDB2^ complex, which consists of CUL4A, RBX1, DDB1, and DDB2. This complex facilitates subsequent detection by XPC. In TC-NER, damage recognition is initiated by stalled RNA Pol II, which recruits various factors, including CSA, CSB, and the CUL4A-RBX1-DDB1-E3 ubiquitin ligase complex (CRL4^CSA^). Both GG-NER and TC-NER pathways converge to recruit TFIIH, which unwinds the DNA after the release of the CAK subcomplex. The damaged segment is then excised by two endonucleases, XPF-ERCC1 and XPG. Once the damaged DNA is removed, DNA resynthesis is carried out by cell-cycle-dependent polymerases and ligases. DNA lesions are schematized by red asterisks, the 5′ incision by XPF-ERCC1 and the 3′ incision by XPG by red arrows.

In eukaryotes, NER is a dynamic process that operates through the sequential assembly of more than 20 proteins at the DNA lesion. NER can be initiated by two sub-pathways, i.e., the global genome NER (GG-NER) or the transcription-coupled NER (TC-NER), which differ in the mechanism of recognition of DNA damage (Figure 2). GG-NER examines the entire genome for helix distortions, while TC-NER is responsible for rapid DNA repair on the transcribed strand of active genes [11,47,48,49,50].

#### 3.1.1. Damage Recognition in GG-NER

During GG-NER, XPC, in association with HR23B and CENT2, mediates lesion recognition by detecting DNA helix distortion [25,26,46]. Structural studies have shown that Rad4, the yeast orthologue of XPC, does not directly interact with DNA lesions. Instead, it detects lesion-induced helical distortions by binding to the undamaged DNA strand and inserting a β-hairpin domain into the DNA duplex. This insertion leads to the formation of single-stranded regions due to the compromised hydrogen bonding between the damaged and undamaged strands [51,52,53]. Biophysical studies that include single-molecule imaging and molecular dynamics simulations provided complementary insights into the mechanism and energetics of lesion recognition by Rad4/XPC. They revealed a two-step recognition process (termed “twist-opening”) [54], during which Rad4-Rad23 performs both random walks and sub-diffusion to facilitate damage recognition [55].

While XPC has a high affinity for 6-4PPs, its binding to CPDs is relatively inefficient. Indeed, CPDs cause only minimal thermodynamic destabilization of the helix compared to 6-4PPs and are predominantly generated in nucleosomal DNA [56,57]. Consequently, efficient detection of CPDs by XPC requires additional factors, particularly the UV-damaged DNA binding (UV-DDB) factor, which consists of the Damage-Specific DNA Binding protein 1 (DDB1), also known as Xeroderma Pigmentosum group E protein (XPE), and 2 (DDB2). This complex is recruited to damaged chromatin and facilitates the subsequent arrival of XPC by repositioning nucleosomes to facilitate access to the lesion and enhancing the distortion of the DNA helix [58,59]. Structures of DDB1-DDB2 complexes bound to damaged DNA reveal that the lesion is exclusively held by the WD40 domain of DDB2. By inserting a hairpin into the minor groove of the damaged DNA, DDB2 extrudes the photodimer into a binding pocket and kinks the duplex. Additionally, DDB2 also stabilizes the opposite strand, inducing a slight unwinding of the DNA around the damaged site [60]. Recent cryo-electron microscopy (Cryo-EM) studies of UV-DDB bound to nucleosomes bearing DNA damage mimics have provided insights into how UV-DDB detects occluded lesions in tightly positioned nucleosomes. These studies identified slide-assisted site exposure as a mechanism that enables high-affinity DNA-binding proteins to access otherwise concealed sites in nucleosomal DNA [61].

The DDB1-DDB2 heterodimer is part of the CRL4 ^DDB2^ E3 ubiquitin ligase complex, which also includes RING-Box Protein 1 (RBX1), Cullin-4A (CUL-4A), and the Constitutive Photomorphogenic 9 (COP9) signalosome. In response to UV irradiation, COP9 dissociates from CRL4 ^DDB2^, activating its ubiquitin ligase activity [62,63]. Activated CRL4 ^DDB2^ substrates include histones H2A, H3, and H4, and their ubiquitination disrupts histone–DNA interactions, facilitating DNA opening [64,65]. Additionally, CRL4 ^DDB2^ catalyzes the auto-ubiquitinylation of the lesion-bound DDB2, promoting its eviction by the p97/Valosin-Containing Protein (VCP) segregase and subsequent degradation by the proteasome [62,63,66]. This complex also reversibly ubiquitylates XPC, which is crucial for efficient damage transfer from DDB2 to XPC [67,68].

#### 3.1.2. Damage Recognition in TC-NER

TC-NER was first discovered in Chinese hamster ovary cells and characterized as the preferential repair of the transcribed strand [69]. After four decades of research, it is now well established that TC-NER is triggered when RNA Pol II encounters a DNA lesion and is unable to past it [70,71]. This blockage is detected by the Cockayne syndrome protein B (CSB) [72], which is mutated in a human neurodegenerative progeroid syndrome called Cockayne syndrome (CS). CSB, the human ortholog of the *Escherichia. coli* Mfd and yeast RAD26 genes, is a SWItch/Sucrose Non-Fermenting (SWI/SNF)-type ATPase that binds to the upstream side of stalled RNA Pol II and attempts to push it past obstacles. While the Mfd protein is both necessary and sufficient for TC-NER in prokaryotes, mammalian cells require additional components [73]. In particular, TC-NER requires the Cockayne syndrome protein A (CSA), which interacts with the Cullin 4-RBX1-DDB1-E3 ubiquitin ligase complex (CRL4^CSA^) [74,75]. This interaction facilitates the recruitment of the UV-stimulated scaffolding protein A (UVSSA) and the elongation factor Elongation Factor 1 (ELOF1) to the stalled elongation complexes [76]. CSA and CSB then work together to promote chromatin decompaction by facilitating the recruitment of chromatin remodelers, such as SWI-SNF [77], and enhancing the ubiquitination of lysine 1268 of the largest subunit of RNA Pol II. This leads to the degradation or eviction of RNA Pol II, facilitating the recruitment of TFIIH [71,78,79]. The recruitment of TFIIH is also facilitated by Serine/Threonine Kinase 19 (STK19), which is recruited on stalled RNA Pol II by CSA and promotes the ubiquitination of UVSSA [80].

Recent advancements have significantly enhanced our understanding of the structural details of TC-NER complexes, particularly through the determination of cryo-EM structures of human RNA Pol II complexes containing CSB, CRL4^CSA^, UVSSA, and ELOF1 [81,82], as well as the Platelet Activating Factor 1 (PAF1) complex and the transcription elongation factor Suppressor of Ty 6 (SPT6) [83]. Together with previous biochemical results, these structures provide a model for transcription–repair coupling, particularly regarding RNA Pol II ubiquitylation and backtracking: when RNA Pol II stalls at a DNA lesion, CSB replaces the elongation factor DRB Sensitivity-Inducing Factor (DSIF), binds PAF1, and moves upstream DNA to SPT6 [82]. In this context, ELOF1 acts as an adaptor and stabilizes the positioning of UVSSA and CRL4^CSA^ on the arrested RNA Pol II. This interaction facilitates the neddylation of the ligase and activates RNA Pol II ubiquitylation. Additionally, ELOF1 enables the recruitment of the transcription factor IIS (TFIIS)-like element, which organizes UVSSA through the RNA Pol II pore, thereby preventing the reactivation of the RNA Pol II [81].

### 3.2. Damage Verification and DNA Opening

Following damage recognition, TFIIH is recruited through its p44 and p62 subunits, coinciding with the eviction of DDB2 from the lesion [84]. During GG-NER, XPC facilitates the recruitment of TFIIH. In contrast, during TC-NER, the CSA/CSB complex, in association with ubiquitinated UVSSA and ELO1, recruits TFIIH [79,85,86]. Recent structural studies characterized the interaction between UVSSA and TFIIH, revealing that TFIIH induces conformational changes in UVSSA, which promote the ubiquitination of CSB by CRL4^CSA^ and facilitate their release [84,87]. TFIIH, through the ATPase and helicase activities of its XPB and XPD subunits, verifies the DNA lesion and unwinds the surrounding region (see Section 5, Focus on TFIIH in NER). This opening occurs over 25 to 34 nucleotides once the cyclin-dependent kinase activating kinase (CAK) subcomplex of TFIIH has been ejected. CAK eviction coincides with the recruitment of Xeroderma Pigmentosum group A protein (XPA) [44,88], a protein involved in lesion verification. XPA also potentiates TFIIH accumulation and DNA opening and promotes the release of XPC [89,90]. The release of XPC is also promoted by Ring Finger Protein 111 (RNF111), which ubiquinates XPC [91]. After DNA opening, Replication Protein A (RPA) (an heterotrimer composed of the RPA14, RPA32, and RPA70 proteins) is recruited to protect the single-strand DNA [92]. RPA also stabilizes the incision complex and contributes to the correct positioning of NER factors, including XPA [93].

### 3.3. Damaged DNA Excision and Resynthesis

Following damage recognition and verification, the XPF-ERCC1 and XPG endonucleases are recruited to cleave the damaged strand. XPF-ERCC1 and XPG respectively cut the DNA at 3′ and 5′ from the lesion at the single-strand/double-strand DNA border, leaving 3′ hydroxyl and 5′ phosphate ends and enabling direct repair without modification of the DNA [94]. Genome-wide analysis of human GG- and TC-NER showed that ERCC1-XPF cleaves the DNA approximately 20 nucleotides (nts) ± 5 nts upstream of the damage site, while XPG cleaves about 6 nts ± 3 nts downstream of the lesion, leading to the removal of a single strand roughly 30 nts long (in the 24 to 32 nts range) [24,95].

Recent structural studies have provided new insights into the mechanisms of action and regulation of the XPG and XPF-ERCC1 endonucleases. In particular, the crystal structure of the XPG catalytic domain responsible for its DNA binding and nuclease activity provided structure-based hypotheses for how XPG recognizes its bubble DNA substrate [96]. Single-molecule studies suggested that XPG only cleaves DNA once TFIIH is blocked at a lesion, and a permissive single-strand DNA length of more than 15 nucleotides is generated [97]. Cryo-EM structures of XPF-ERCC1 revealed the overall architecture of the complex and uncovered a previously unreported autoregulatory mechanism. The XPF–ERCC1 complex was shown to adopt an autoinhibited conformer in the absence of DNA in order to prevent promiscuous cleavage, providing structural evidence for the initial steps of XPF–ERCC1 activation upon binding a DNA junction [98]. So far, structural data that can explain how the XPG or XPF-ERCC1 endonucleases are recruited on the pre-incision complex with TFIIH are still lacking. However, in archaea, the crystal structure of the XPB helicase in association with Bax1, an XPG-like nuclease, and a DNA substrate have been obtained, providing new insights into the molecular mechanisms of XPB-mediated DNA repair bubble formation in archaeal and eukaryotic NER [99].

After excision, the oligonucleotide containing the lesion bound to TFIIH is released. A recent study showed that the helicase-like transcription factor (HLTF) plays a crucial role in NER by actively evicting damaged DNA [100]. Specifically, HLTF mediates the transition between DNA excision and synthesis by promoting dissociation of the damage-stably bound incision complex and the incised oligonucleotide. This activity promotes the recruitment of Proliferating Cell Nuclear Antigen (PCNA), whose association with DNA is regulated by the clamp loader complexes Replication Factor C (RFC) and Chromosome Transmission Fidelity Factor 18 (CTF18)-RFC, contributing to repair synthesis. The damaged DNA fragment bound to TFIIH is then processed and degraded by the 3′-5′ exonuclease Three prime Repair Exonuclease 1 (TREX1) and possibly another unidentified nuclease [101].

Following the removal of the lesion-containing oligonucleotide, the DNA resynthesis system occurs, depending on the cell cycle stage. In interphase, the excised portion is resynthesized by the DNA polymerases δ and κ, while during mitosis, DNA polymerase ε is required. The neosynthesized oligonucleotide is ligated either by Ligase 3 and X-ray Repair Cross-Complementing 1 (XRCC1) in interphase or by Ligase 1 and Flap Endonuclease 1 (FEN1) endonuclease in dividing cells [47]. Further studies confirm that DNA resynthesis can occur with all three DNA polymerases, i.e., δ, ε, and κ, depending on the ubiquitination state of PCNA (Proliferating Cell Nuclear Antigen) and XRCC1 [102]. Approximately 50% of repair synthesis is carried out by DNA polymerase κ, which is recruited by ubiquitinated PCNA and XRCC1, along with DNA polymerase δ, which is recruited by the classical RFC complex. The remaining 50% is performed by DNA polymerase ε, which is recruited by the CTF18-RFC complex [102].

## 4. Composition and Structure of TFIIH

**Figure 3:**
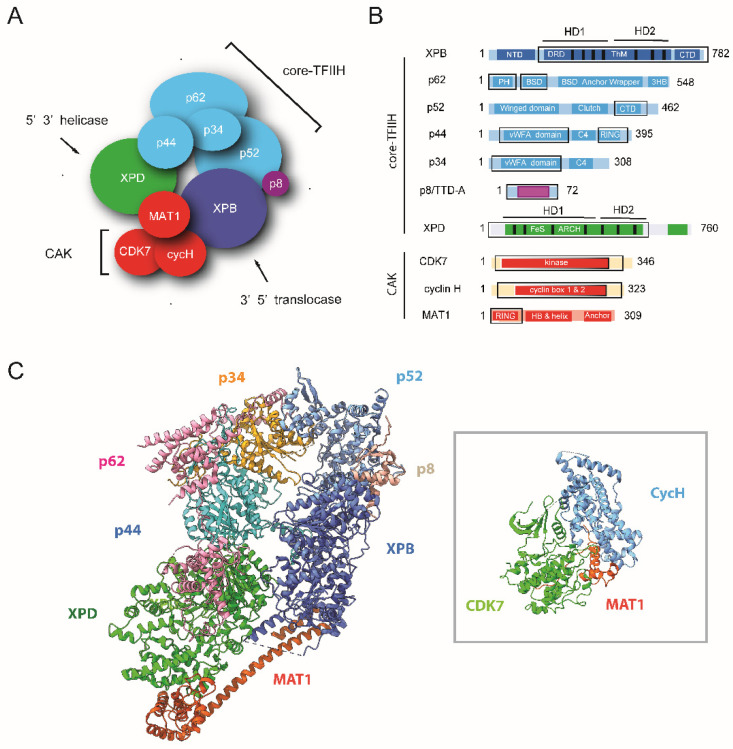
Architecture of human TFIIH. (**A**) TFIIH consists of 10 subunits organized into two structural and functional entities: (i) core-TFIIH, which includes the XPB translocase (in blue), the XPD helicase (in green), and the regulatory/scaffolding subunits p8/TTD-A, p34, p44, p52, and p62 (in magenta and cyan), and (ii) CAK, a heterotrimeric kinase complex made up of CDK7 kinase, cyclin H, and MAT1 (all in red). (**B**) Schematic representation of the 10 TFIIH subunits, highlighting their domain organization. The RecA-like helicase domain (HD) and helicase motifs (black bars) of XPB and XPD are shown. Black boxes indicate modules whose structures have been determined by X-ray crystallography or NMR. (**C**) Ribbon representation of core-TFIIH, including the N-terminal region of MAT1, from the cryo-EM structure of Holo-TFIIH (PDB ID 6NMI) [107]. The structure of CAK, which is not visible in 3D reconstructions of Holo-TFIIH, is shown in the insert (PDB ID 8PLZ) [108].

Human TFIIH has a total molecular weight of approximately 500 kDa and can be divided into two functional subcomplexes: the seven-subunit core-TFIIH, which contains the XPB and XPD helicases, and the heterotrimeric CAK, comprising the CDK7 kinase (Figure 3A). While XPB- and XPD-like proteins are detected in certain bacteria, many archaea, and universally in eukaryotes, the other TFIIH subunits p62, p52, p44, p34, and p8/TTD-A (also termed GTF21-5) are exclusively present in eukaryotes. Sequence identities between human and yeast TFIIH subunits range between 26% and 56%, the highest conservation being observed for the catalytic subunits XPB, XPD, or CDK7. On the contrary, the non-enzymatic subunits p8/TTD-A, p34, p52, and particularly p62 have significantly diverged or are undetectable [103,104]. Transcriptionally active TFIIH of the early-diverged eukaryote *Trypanosoma brucei* (*T. brucei*) harbors two novel core subunits but not a cyclin-activating kinase complex, which is consistent with the absence of the YSPTSPS motif in the CTD of the largest subunit of RNA Pol II [105]. Interestingly, *T. brucei* possesses a second XPB paralog that does not associate with other TFIIH subunits but instead plays a specialized role in NER [106].

### 4.1. The Seven-Subunit Core-TFIIH

The seven-subunit core-TFIIH is composed of the XPB translocase, the XPD helicase, and five non-catalytic subunits: p62, p52, p44, p34, and p8/TTD-A. These TFIIH non-catalytic subunits participate in the intricate network of protein–protein interactions required for the assembly of the TFIIH core complex and the regulation of XPB and XPD (Figure 3B,C).

XPB and XPD, the two largest core-TFIIH subunits, belong to the SF2 family of DNA-dependent ATPases. Both proteins are built around a central catalytic core composed of two RecA-like motor domains (HD1 and HD2) characterized by seven conserved catalytic motifs [109,110]. The crystal structure of an archaeal homolog of human XPB revealed the existence of a damage recognition domain (DRD) containing a specific RED motif and of a DNA polymerase thumb-like motif (ThM) [111]; both are involved in the catalytic and repair activity of XPB [99,112]. The central catalytic core of XPB is flanked by a highly conserved N-terminal domain of 20 kDa (NTD), which specifically interacts with the p52 TFIIH subunit [113] and C-terminal extension, whose phosphorylation controls the incision by the ERCC1-XPF endonuclease [114]. XPB activity is required for both transcription initiation and DNA repair. Although XPB—as well as its Ssl2 yeast orthologue—was identified as a TFIIH subunit with DNA helicase activity [36], it is now well established that this protein is a DNA translocase. In vitro experiments clearly established that the yeast–TFIIH complex exhibits XPB/Ssl2-dependent dsDNA translocase activity and primarily tracks along one DNA strand in the 5′-3′ direction [115,116,117]. Human TFIIH possesses weak intrinsic translocase activity, which is stimulated by XPA [118].

XPD possesses 5′ 3′ helicase activity, which is dispensable for the transcription activity of TFIIH but is absolutely required for DNA repair [119]. As will be further discussed, the helicase activity of XPD is stimulated by an interaction with the p44 core-TFIIH subunit [120] and inhibited by MAT1 [121]. Structures of XPD archaeal homologs revealed that in addition to the two canonical helicase HD1 and HD2 domains, the protein is composed of a 4FeS cluster domain involved in DNA damage recognition and of a novel domain, termed the ARCH domain (as its shape is reminiscent to that of an arch) [122,123,124]. A C-terminal extension, conserved among eukaryotic XPDs but absent in archaeal homologs, is responsible for the interaction with the p44 core-TFIIH subunit and the regulation of its helicase activity [125].

**Figure 4:**
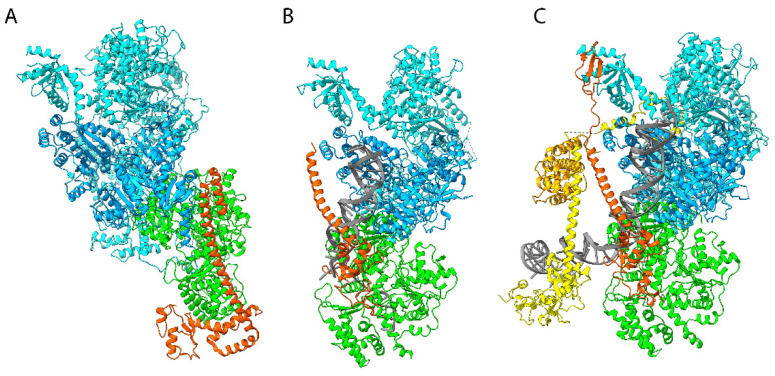
Structures of TFIIH in different functional states. (**A**) Core-TFIIH with the N-terminal part of MAT1 (PDB ID 6NMI) [127]. (**B**) Human core-TFIIH complexed to XPA and branched DNA (PDB ID 6RO4) [118]. (**C**) Complex between core-TFIIH, XPA, XPC, and damaged DNA (PDB ID 8EBX) [119]. All structures are superimposed on XPB to illustrate the displacement of XPD after XPA binding. TFIIH core subunits are colored in cyan, with the exception of XPB in blue and XPD in green. MAT1 and XPA are shown in red, and XPC is shown in yellow.

Building on atomic structures of isolated subunits and domains obtained by X-ray crystallography and nuclear magnetic resonance (NMR), cryo-EM structures of TFIIH in different functional states were determined. They include the structure of the human core complex, several structures of TFIIH associated with RNA polymerase II as well as core-TFIIH associated with the XPA NER factor and a DNA substrate or with XPA, XPC and a damaged DNA fragment (Figure 3 and Figure 4) [107,118,119,126,127,128]. These structures revealed that the architecture of core-TFIIH is dominated by the XPB and XPD subunits, which are situated next to each other at the open end of the horseshoe-shaped structure. The remaining subunits form a semicircular arrangement, sequentially comprising p52, p34, and p44, with the p8 subunit linked to p52. The p62 subunit extends along the horseshoe-shaped surface, interacting with p34, p44, and XPD (Figure 3).

The cryo-EM structures of XPD showed the expected domain arrangement of the RecA-like domains, with insertions of the 4FeS cluster (FeS) and ARCH domain. These insertions create a central pore through which DNA passes before interacting with the helicase motifs [107]. The structure of a TFIIH-XPA-DNA complex [118] revealed that that single-stranded DNA extends towards XPD, contacts the DNA-binding motifs of the helicase domains of XPD, and then stretches across the XPD pore, as predicted from structures of archaeal homologs [122,123,124] and as observed in DinG, a DNA-bound bacterial XPD homolog [129].

The structures of core-TFIIH provided detailed insights into the intricate network of protein–protein interactions required for the assembly of the TFIIH core complex and the regulation of its catalytic activities. Among them are the interaction between the C-terminal domain of XPD (CTD) and the N-terminal von Willebrand factor A (vWFA) domain of p44, which positively regulates helicase activity [120,126,130]. The p44 subunit, whose C-terminal region is composed of a C4 zinc finger (C4) followed by a RING domain with two interleaved zinc binding sites (RING) [131,132], tightly interacts with p34 through both its N-terminal vWFA domain and its C-terminal C4 zinc finger (C4) [133]. These interactions form a multivalent network that is further stabilized by interactions between the vWFA domain of p34 and the N-terminal winged helical domain (Winged) of p52, as well as with the C-terminal three-helix bundle (3HB) of p62. Interestingly, the N-terminal PH domain of p62, which mediates interactions with components of the core transcription and NER machinery [134,135], is disordered in the structure of free TFIIH, while the remainder of the protein is almost completely resolved and exhibits a beads-on-a-string topology. This arrangement includes 2 BSD domains that are characterized by the presence of a conserved FWxxΦΦ motif (Φ representing either tyrosine or phenylalanine) followed by two regions known as the Anchor and Wrapper and a C-terminal three-helix bundle (3HB) [107]. In agreement with previous biochemical and structural studies [136,137,138], and of potential importance for the regulation of XPD helicase activity, the structure of free TFIIH [107] revealed that the central region of p62 interacts with the second RecA-like motor domain (HD2) of XPD.

Finally, cryo-EM data also provide a structural basis for the recruitment of XPB by p52 and for the regulatory interactions that control XPB ATPase activity [113,125]. Consistent with biochemical data from reconstituted in vitro systems [113], the structure of the TFIIH core complex [107] revealed how the recruitment of XPB by p52 depends on a pseudo-symmetric dimer of homologous domains in these two proteins—the XPB N-terminal domain (NTD) and the clutch domain of p52 (Clutch). This interaction is stabilized by the p8/TTD-A subunit, which is absolutely required for the DNA repair activity of TFIIH [114] and tightly interacts with p52. Strikingly, p8/TTD-A and the p8/TTD-A binding domain of p52 also adopt the same fold and form a pseudo-symmetric heterodimer [139,140].

### 4.2. The CAK Heterotrimer

CAK is a heterotrimeric complex composed of cyclin-dependent kinase 7 (CDK7), cyclin H, and the ménage à trois 1 protein (MAT1) (Figure 3A,B), which exists as a free entity associated with the XPD helicase or as part of TFIIH [141]. As part of TFIIH, CAK hyper phosphorylates the C-terminal heptapeptide repeat region of the largest subunit of RNA polymerase II (Pol II), a key event in the regulation transcription of eukaryotic protein-coding genes, which leads to promoter clearance [142]. In addition to its direct involvement in transcription via TFIIH, CDK7 is also an activating kinase for the transcription elongation kinases CDK9, CDK12, CDK13, and probably CDK11 [143]. As discussed below, pharmacologic inhibition of CDK7 has emerged as a promising option for cancer treatment (see Section 9, Is TFIIH an Attractive Drug Target?).

While X-ray crystal structures of CDK7 and cyclin H provided the first insights into the specificity and its regulation by its associated cyclin [144,145,146], structural information on the three-subunit CAK remained elusive until experimental structures of the ternary complex were determined (Figure 3C) [147,148,149]. These structures, in particular, provide the molecular basis to understand how the C-terminal region of MAT1, known to bind the CDK7/Cyclin H complex and to activate CDK7 kinase activity [150], substantially extends the interaction interface between CDK7 and cyclin H, explaining its role as a CAK assembly factor. The recent high-resolution structures of CAK complexed with small-molecule inhibitors pave the way to rationally optimize design CDK7 inhibitors and develop new therapeutic strategies [108]. While the mechanistic basis of CAK substrate specificity is currently not clear, a recent X-ray crystal structure of human CAK provided a structural basis of CDK7 activation by dual T-loop phosphorylation and showed, in particular, that phosphorylated S164 participates in an interaction network involving residues from cyclin H and MAT1 [151].

MAT1 not only functions as an assembly factor that stabilizes and activates the CDK7/cyclin H pair but also acts as a bridging subunit linking CAK to core-TFIIH. Specifically, the cryo-EM structure of free human TFIIH revealed that the tethering of CAK to core-TFIIH relies on a three α-helix bundle and on a long α-helix (HB and helix), which directly interact with the XPB DRD-like domain and with the XPD ARCH domain. The structure describes a number of inhibitory interactions that block ATP and DNA binding sites in XPD. Comparisons of the free TFIIH structure with those of the complex engaged in NER where MAT1 is absent [118,119] revealed significant conformational rearrangement that can explain TFIIH activation in the context of DNA repair.

## 5. Focus on TFIIH in NER

During NER, TFIIH primarily acts as a helicase to unwind DNA in the vicinity of the lesion, verifies the presence of damage, and keeps the DNA bubble open, enabling the recruitment of downstream repair proteins [50,152]. The recruitment of TFIIH relies on XPC during GG-NER and involves UVSSA during TC-NER or as illustrated in the previous section (also see Section 3, The NER Pathways).

Structural studies have uncovered the molecular basis of TFIIH recruitment by XPC during GG-NER. In particular, cryo-EM structures of yeast TFIIH in association with XPC on damaged DNA revealed that XPB, through its R-E-D and Thumb domain, first interacts with DNA on the 5′ side of the lesion, while XPC interacts with the 3′ part. XPB then uses its ATPase activity to induce torsion and unwind the DNA, while XPC prevents free DNA rotation [85]. These studies also suggested that once TFIIH is anchored to the DNA, XPD interacts with the DNA, first through the helicase domain 2 (HD2) and then through a second binding site on helicase domain 1 (HD1). Consistent with previous studies [150,153,154], this positioning permits DNA entry into the XPD pore, enabling the lesion’s verification as well as DNA opening. Since the complete pre-incision complex has not yet been fully obtained, recent prediction models based on cryo-EM, XL-MS, and AlphaFold2 have provided valuable insights. These models suggest that TFIIH positioning also acts as a molecular ruler, determining the size of the DNA bubble and precisely positioning the XPG and XPF nucleases for incision [155].

The recruitment and stabilization of core-TFIIH have subsequently been detailed in living cells, revealing that other factors contribute to the stabilization of the complex on damaged DNA. Indeed, XPA promotes the activity as well as the stabilization of TFIIH to the DNA lesion, while ERCC1-XPF and XPG facilitate its dissociation [97,156,157]. The depletion of XPG leads to TFIIH persistence at damaged sites, preventing proper damage removal [157]. This persistence, characterized by greater immobilization of the complex on chromatin up to three hours after UV irradiation, is associated with senescence in human cells and in *Caenorhabditis elegans*. In contrast, the depletion of XPA results in a lower immobile fraction of TFIIH both immediately and three hours after UV irradiation, confirming a rapid dissociation of TFIIH upon XPA depletion [157]. Recent structural analyses confirm these observations, showing that XPA, which binds between XPB and XPD, induces a kink in the DNA duplex and shifts both XPC and the DNA lesion by nearly one helical turn relative to core-TFIIH. As a result, the DNA lesion is positioned outside of core-TFIIH, similar to the positioning observed with RNA polymerase (detailed below). XPB and XPD, which track the lesion-containing strand but translocate DNA in opposite directions, work together to push and pull the lesion-containing strand into XPD for verification [119].

Other factors are also known to promote TFIIH anchoring on DNA lesions, notably DDB2, whose overexpression leads to an increased immobilized fraction of TFIIH [158]. Moreover, while XPB is necessary and sufficient in vitro for the recruitment of TFIIH to the lesion, it has been demonstrated in live cells that mutations in XPD lead to TFIIH detachment from the lesion. Indeed, fluorescence loss in photobleaching (FLIP) approaches revealed that in cells expressing the catalytically inactive K48R XPD mutant, TFIIH dissociates from the DNA lesion faster compared to the wild-type, suggesting that XPD’s helicase activity is crucial for forming a stable complex at the lesion. Similar results were obtained with the Y192A and R196E mutations (located in XPD’s DNA entry tunnel), illustrating that some mutations may not affect helicase and/or ATPase activity but impair the ability of XPD to confirm the presence of DNA lesions [159].

Strand-specificity, as well as lesion verification by XPD, is enabled by single-stranded DNA crossing through a pore formed by the iron–sulfur cluster and the ARCH domain [160]. Indeed, DNA progression through this pore is stopped when a lesion is present, blocking TFIIH precisely at the site of the lesion [159]. Structural analyses also showed that p44 plays a significant role in determining the presence of the lesion by a direct interaction. In vitro assays also showed that p44 stimulates the ability of XPD to be bound to photodamage [161] and that the p44–p62 subcomplex interacts with damaged DNA even in the absence of XPD [162]. The recent cryo-EM structure of an XPD–p44–p62 complex from *Chaetomium thermophilum*, determined in the presence of a Y-shaped DNA fork, highlighted the role of the ARCH domain in the active separation of double-stranded DNA [163].

The seven-subunit core-TFIIH supports the 5′-3′ helicase/ATPase activity of XPD which is stimulated by p44 and inhibited by the MAT1 subunit of CAK [43,120,121,164]. This explains why the eviction of CAK from core-TFIIH by XPA is absolutely required to promote DNA opening around the lesion site [88]. Structural studies revealed that the N-terminal domain of MAT1 interacts with the ARCH domain of XPD, immobilizing XPD in a configuration where the single-stranded DNA entry pore is obstructed. Following the recruitment of XPA, CAK removal allows for conformational changes in XPD that unlock the pore and permit single-stranded DNA entry, thus activating its helicase function [118,149].

The mechanism of action of TFIIH during TC-NER is not understood as well as in GG-NER, although it is supposed to be similar [165]. Recent studies outlined the stepwise assembly of TC-NER factors CSB, CSA, UVSSA, and TFIIH around lesion-stalled RNA Pol II. Since the removal of RNA Pol II is essential for allowing repair proteins to access the DNA lesion, these studies identify STK19 as a key factor that facilitates the transition from recognition to downstream repair steps. Indeed, the loss of STK19 delays the clearance of lesion-stalled RNA Pol II, impairing subsequent repair processes. Cryo-EM and mutational analysis reveal that STK19 associates with the TC-NER complex, positioning itself between RNA Pol II, UVSSA, and CSA, and helps position the ATPase subunits of TFIIH onto the DNA in front of RNA Pol II [166,167].

## 6. TFIIH in Transcription Initiation

During the transcription of class II genes, encoding messenger RNAs (mRNAs), nuclear RNAs, and microRNAs, TFIIH enters in the composition of the PIC, composed of TFII-A, -D, -B, -E, and -F and RNA Pol II [30,128,168]. TFIIH interacts directly with DNA and participates in the opening of the DNA double helix around the promoter, thanks to the translocase activity carried by XPB, as well as the phosphorylation of various transcription factors by its kinase activity [117,169]. CDK7 phosphorylates Ser5 and Ser7 in the heptapeptide repeats (Tyr1-Ser2-Pro3-Thr4-Ser5-Pro6-Ser7) of the C-terminal domain (CTD) of the largest subunit of the RNA Pol II subunit B1 (RPB1).

TFIIH is, therefore, involved in two key steps of transcription initiation: DNA opening and promoter escape through its enzymatic subunits XPB and CDK7. XPB was initially described as a helicase that directly opens DNA around the promoter; however, in vitro analyses showed that XPB does not possess 3′ 5′ helicase activity but is an ATP-dependent translocase that is controlled by p52 and p8 [125]. Those analyses also showed that transcription can occur in the absence of XPB and, above all, that TFIIH destabilizes the PIC [170,171]. These results suggest a model in which XPB prevents promoter opening during the sequential assembly of transcription factors on the promoter. Following PIC formation, XPB, through its ATP-dependent translocase activity, moves upstream of the transcription start site, allowing DNA opening around the promoter [170].

Transcript initiation also depends on the RNA Pol II CTD phosphorylation state. In particular, CDK7 phosphorylates CTD on serine 5 and 7 of the CTD at the beginning of transcription, which, after PIC assembly and promoter melting, leads to the escape of RNA Pol II from the promoter and triggers transcription elongation [172,173]. Phosphorylation of serine 5 also enables the recruitment and regulation of numerous factors involved in biological events such as RNA Pol II pausing and post-transcriptional mRNA modifications [174,175]. CDK7 also phosphorylates the CTD serine 7, which plays a role in the interaction with mRNA maturation machinery, notably, proteins involved in the capping and splicing of pre-mRNAs into mRNA. The phosphorylation of serine 7 also weakens interactions between RNA Pol II and PIC, leading to RNA Pol II escape from the promoter [176,177]. Recent biochemical analysis revealed an unexpected dynamic process during which TFIIEα and TFIIEβ act as key factors to recruit the CAK of TFIIH within the PIC and showed that RNA Pol II phosphorylation is accompanied by the release of CAK and TFIIEα from the promoter [178].

The structure of PIC complexes has been an area of intense study in recent years, in particular, on naked core promoters. Structural data obtained in the presence of the Mediator complex with proteins isolated from Saccharomyces cerevisiae and humans [136] revealed details of the mechanism of TFIIH recruitment into the PIC. These structures visualize that TFIIH interacts directly with the minor grooves of the DNA helix, downstream of the promoter (25–30 bases) through XPB, and that the two enzymatic domains are located 40-A from the DNA, confirming that XPB enzymatic activity is not required during the early stage of transcription. Data also show how CAK interacts with the Mediator complex (via MAT1) and how it orientates CDK7 to phosphorylate the RNA Pol II CTD [179]. Indeed, the structure shows that TFIIE interacts with three sites of TFIIH (the PHD and BSD1/2 domains of p62 and the ARCH domain of XPD), providing a platform for TFIIH to interact with RNA Pol II and explaining why TFIIE is necessary for TFIIH recruitment [136,178].

Mutations and transcriptional deregulation of several global genome-organizing complexes are linked to global alterations in the chromatin structure and have emerged as key players in human diseases [180]. Some histone acetyl-transferase (HAT) complexes not only carry out gene-specific regulatory functions but also exhibit global chromatin-modifying functions by regulating higher chromatin organization. Recent analyses revealed that TFIIH, by interacting functionally with KAT2A, an acetyl-transferase that belongs to the hSAGA and hATAC complexes, elicits large-scale chromatin decondensation [181]. In XP-B/CS cells, the abnormal recruitment of TFIIH and KAT2A to chromatin causes the inappropriate acetylation of histone H3K9, leading to the aberrant formation of transcription initiation complexes on the promoters of several hundred genes and their subsequent overexpression.

## 7. Insights into the Non-Canonical Roles of TFIIH

**Figure 5:**
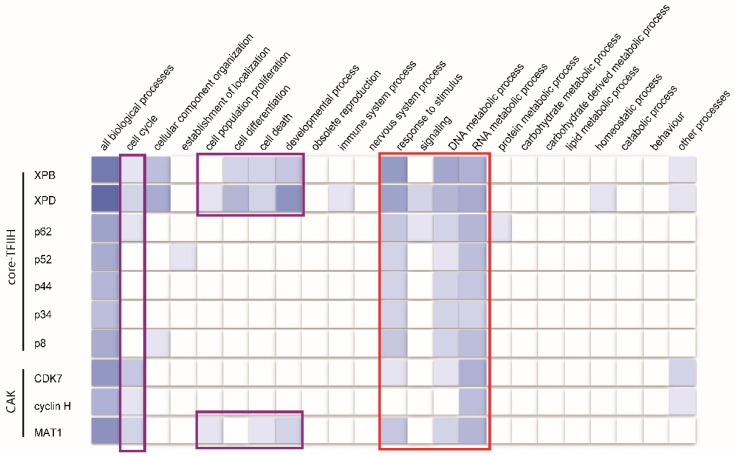
Gene Ontology (GO) of TFIIH molecular functions. The molecular functions of the genes encoding the 10 TFIIH subunits are visualized using the Gene Ontology (GO) Ribbon https://geneontology.org/ (accessed on 30 January 2025). The color intensity in the ribbon represents the confidence or strength of the molecular function annotations: dark blue indicates well-established functions, while white reflects functions that are less understood or more variable. Annotations that broadly reflect TFIIH’s roles in DNA repair and transcription are highlighted within the red box, while those linking TFIIH to cell cycle-related events and developmental processes are highlighted in magenta.

Beyond its well-known roles in transcription and DNA repair, TFIIH and its subunits are also involved in additional cellular processes, as highlighted by a Gene Ontology (GO) analysis of the genes encoding TFIIH subunits (Figure 5). In addition to a clear participation in DNA and RNA metabolic processes, as well as cellular responses to stimuli, annotations also suggest the involvement of the CAK subcomplex, and the XPB and XPD core subunits, in cell cycle regulation. These findings further show that TFIIH is linked to a variety of other cellular functions (Figure 5).

Notably, TFIIH has been observed at telomeres [182], where it participates in their replication [183]. Indeed, a recent study revealed that XPB, p52, and p34 interact with Telomeric Repeat-binding Factor 1 (TRF1), a pivotal component of the Shelterin complex responsible for binding telomeric DNA and playing a crucial role in telomere maintenance and replication [183]. Evidence supporting a role of TFIIH in telomere replication comes from a study showing that the depletion of TFIIH subunits leads to telomere replication defects comparable to those observed following TRF1 deletion replication [183]. Moreover, a TRF1 mutant unable to interact with TFIIH results in severe telomere replication defects, mirroring the phenotype associated with TFIIH dysfunction. This highlights a potential functional connection between TFIIH and telomere replication pathways, although the exact mechanisms are still not fully understood.

Furthermore, certain subunits of the TFIIH complex, specifically the CAK subcomplex and XPD, are involved in the regulation of the cell cycle in a manner that is independent of TFIIH [40,141,184,185,186,187]. Indeed, the CAK subcomplex was initially identified as a crucial factor in cell cycle regulation, where it activates and adjusts the binding affinities of cyclins to various cell cycle CDKs (CDK1, CDK2, CDK4, and CDK6) by phosphorylating specific threonines, a process called T-loop activation [184,188]. This process induces conformational changes that stabilize the activated form of CDK. Several factors have been shown to regulate CAK kinase activity. In particular, XPD has been shown to negatively regulate CAK activity in Drosophila; excess XPD decreases phosphorylation of the CDK T-loop, leading to mitotic defects, while a lower amount of XPD increases CAK kinase activity and cell proliferation [141]. The role of XPD during mitosis is not restricted to its regulatory functions of CAK kinase activity. Indeed, more recently, mutations in XPD have been shown to affect chromosome segregation in Drosophila and human cells [141,185,187]. To date, the role of XPD in mitosis remains unclear, although it has been shown that XPD interacts with the Maintenance of Mitotic Stability 19 protein (MMS19), a protein necessary for CDK7 kinase activity [186], and with the Mitotic Interactor and Partner of Mms19 18 protein (MIP18) in a complex called MMXD [185]. XPD has also been recently shown to interact with the kinesin 5 (Eg5) on mitotic spindles and midbodies, and this interaction is influenced by phosphorylation events such as the phosphorylation of XPD by the never in mitosis gene A-related kinase 6 (NEK6) at threonine 425 [187]. The same study also showed that mutations in XPD affect its interaction with Eg5 and lead to severe mitotic defects, including misaligned chromosomes and chromatin bridges.

XPB also appears to play a role in the cell cycle, as it has been shown to localize to the centrosomes during chromosome division [189]. To date, the function of XPB in mitosis remains unclear, but it has been shown that it interacts with tubulin, and mutations in XPB lead to defective chromosome segregation. Moreover, XPB’s translocase activity seems to be important in chromatin condensation [190,191]. Indeed, TFIIH has been shown to be crucial for the establishment and maintenance of mitotic chromosome condensation in Xenopus eggs. Notably, research has shown that inhibiting the ATPase activity of XPB, a subunit of TFIIH, disrupts chromosome condensation. This results in effects akin to those observed when condensin, the protein complex responsible for chromosome maintenance, is depleted. Furthermore, TFIIH has been implicated in promoting the enrichment of condensin on chromosomes, highlighting its multifaceted role in chromosomal dynamics during mitosis [191].

Finally, recent studies highlight the involvement of XPD and potentially other TFIIH subunits in mitochondrial genome maintenance after oxidative stress [192]. Indeed, following oxidative stress, XPD relocates to mitochondria and interacts with TUFM (Tu translation elongation factor), a mitochondrial protein. Although the precise role of XPD and the involvement of other TFIIH subunits remain unclear, studies have shown that XPD-deficient cells exhibit a reduced capacity to repair oxidative damage in mtDNA. This leads to increased ROS-associated mtDNA damage and subsequent mitochondrial dysfunction [193].

## 8. The Molecular Basis of TFIIH-Associated Diseases

The significance of TFIIH and its interactions with other NER and transcription factors is underscored by the presence of human genetic disorders that present with a wide range of clinical features. Notably, mutations in the XPB (MIM 133510), XPD (MIM 126340) and GTF2H5 (encoding the p8/TTDA protein, (MIM 608780)) genes are mainly associated with rare autosomal recessive disorders such as Xeroderma pigmentosum (XP), the combination of XP with Cockayne syndrome (XP/CS), and Trichothiodystrophy (TTD). An extensive list of patients affected by these diseases, including amino acid changes, genomic mutations, and their main clinical features, can be found in the following review: [194].

**Figure 6:**
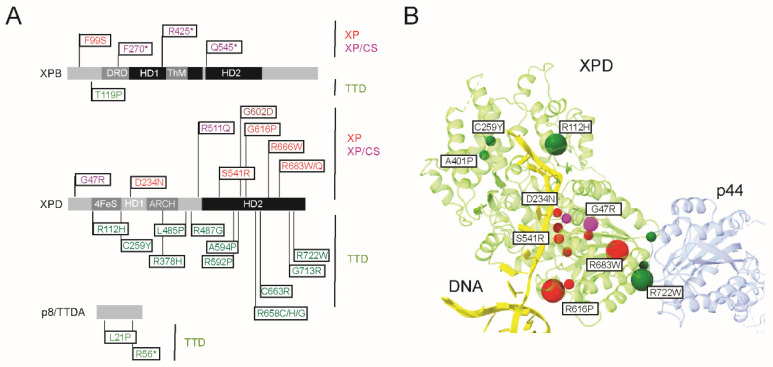
TFIIH mutations and genetic disorders. (**A**) Schematic representation of the XPB, XPD, and p8/TTD-A TFIIH subunits. XP (red), XP/CS (magenta), and TTD (green) mutations are indicated above (XP and XP/CS) or below the sequence. (**B**) Selected XP, XP/CS, and TTD mutations identified in XPD patients are mapped as solid spheres on the structure of human XPD determined in the presence of a DNA substrate (PDB ID 6RO4) [118]. The mutations frequently observed are indicated by large spheres.

With only a few reported cases, mutations in the TFIIH XPB subunit are scarce, which probably reflects the essential role of this subunit in both transcription and DNA repair. In comparison, mutations in XPD have been observed with relatively high frequency, with more than 100 patients [195]. Commonly observed XP, XP/CS, and TTD mutations mapped on the sequences of the XPB, XPD, and p8/TTD-A sequences are listed in Figure 6A.

### 8.1. XP and XP/CS

Xeroderma Pigmentosum (XP) is a rare autosomal recessive genetic disorder characterized by photosensitivity associated with a high predisposition to skin cancers (2000 times higher than in normal individuals). Additionally, around 25% of patients present neurodegeneration due to the progressive loss of neuronal cells [195,196]. Mutations in the XPB and XPD genes, unlike those in DDB2 or XPC that lead to milder forms of XP, are associated with more severe manifestations, including pronounced skin abnormalities, eye lesions, and hyperpigmentation [197,198]. These mutations can cause pure XP cases, typically with minimal neurological defects, or may present in conjunction with other phenotypic features. The Xeroderma Pigmentosum–Cockayne syndrome complex (XP/CS) combines clinical features of XP with those of the CS, which is characterized by neurological and developmental impairments, along with features of premature aging, and is often categorized as a TC-NER disorder [199].

Defects in NER, and, in particular, dysfunctions of the GG-NER pathway, explain the accumulation of DNA damage, genomic instability, and promptness to cancer in XP patients. Neurological symptoms and developmental symptoms, which, in certain cases, are linked to CS, likely result from defects in TC-NER or in transcription, as TFIIH is involved in both processes. By bridging the CAK kinase complex, which plays a key role in transcription, XPB and XPD influence CDK7 activity and, in turn, gene expression. For example, XP-causing mutations, such as XPB T119P or XPD G602D, which have no detectable effects on transcription in vitro, impair the expression of the RARβ2 gene in cells expressing the same variants [200].

### 8.2. Trichothiodystrophy

Trichothiodystrophy (TTD) is a rare autosomal recessive disease characterized by sulfur-deficient brittle hair and nails caused by greatly reduced cysteine-rich matrix proteins [201,202,203]. Symptoms also include intellectual disability, ichthyosis and, in the most severe forms, dwarfism, mental retardation, lipodystrophy, and neurodemyelination. TTD patients with mutations in the XPB, XPD, and p8/TTD-A subunits are all photosensitive but without predisposition to skin cancer [204].

Apart from photosensitivity, these symptoms cannot be explained solely by a DNA repair defect but rather reflect a deregulation of gene expression, as mutations in non-TFIIH subunits that cause TTD lead to similar symptoms. The identification of mutations in the GTF2E2 gene, which encodes the β subunit of the TFIIE general transcription factor from individuals with DNA Repair-Proficient TTD, supports the theory that TTD is caused by transcriptional impairments that are distinct from the NER disorder XP [205]. Cells derived from TTD-causative mutations in TFIIH genes exhibit a reduced NER efficiency with low UV-induced DNA repair synthesis and low intracellular TFIIH concentration, which likely results in an impairment of the transcriptional program [195,206]. In line with this hypothesis, the amount of type VI collagen α 1 (COL6A1)—a ubiquitous component of connective tissues in the dermis—is severely reduced in the skin fibroblasts of TTD XPD R112H, C259Y, and R722W patients. This alteration occurs specifically in TTD cells carrying mutations in XPD and results from a transcriptional defect that is corrected by the expression of wild-type XPD [207]. The analysis of novel XPD thermolabile variants established the direct relationship between quantitative variations in TFIIH cellular content and its functional activities, strongly supporting the link between TFIIH amount and TTD symptoms [208].

TTD patients carrying a mutation in the p8/TTD-A gene exhibit a relatively mild TTD phenotype. In cells isolated from TTD-A patients (carrying mutations in p8) and the TTD-A knockout mouse model (complete deletion of the p8 gene), both transcription and DNA repair activities are severely affected, which may be explained by dramatically reduced steady-state levels of TFIIH. In these cells, repair is not completely abolished but progresses slowly, which also explains their low sensitivity to UV light [194,203]. Additionally, mutations in the p8/TTD-A subunit also affect the binding of TFIIH to rRNA, impacting ribosome biogenesis, notably, the maturation of pre-mRNAs into mRNAs, leading to neurological disorders [209].

A significant contribution to our understanding of the genotype–phenotype relationships is provided by structural interpretation [118,127,210] (Figure 6B). Many XP-associated mutations in the XPD TFIIH subunit map the conserved helicases motifs that constitute the ATP binding site or play a key role in DNA recognition [122,123,130]. This is the case of the G47R and D234N mutations that play a key role in ATP hydrolysis and, thus, in XPD helicase activity in vitro. Residues R616 and R683, frequently mutated not only in XP patients but also in a few XP/CS or TTD patients, are critical for the association between XPD and p44 and, thus, for XPD helicase activity [130]. The XPD R683W allele, which causes XP, not only disrupts the activation of XPD helicase activity but also impairs CAK’s ability to phosphorylate the RNA Pol II CTD, thereby hindering promoter escape. Structural data on the p8/TTD-A subunit revealed how p8/TTD-A stabilizes TFIIH, particularly explaining why mutations in p8 (L21P and R56Stop), which cause TTD-A, weaken its interaction with p52 [139] and impair the stimulation of XPB ATPase activity [128,211]. This leads to a reduction in intracellular TFIIH levels and results in NER defects.

## 9. Is TFIIH an Attractive Drug Target?

Inhibiting DNA repair is an effective strategy to combat cancer, particularly when used to sensitize tumors to DNA-damaging therapies or to exploit synthetic lethality [212]. Olaparib, an approved PARP inhibitor that exploits synthetic lethality in tumors with homologous recombination deficiency, and Berzosertib, which inhibits ATR, a key regulator of the DNA damage response and replication stress pathways, underscore the potential of DNA repair inhibition in oncology.

While there are currently no approved drugs that directly target the NER pathway, several drug candidates that interfere with NER factors and, in particular, TFIIH subunits have been identified. Among these, Triptolide, derived from the traditional Chinese medicinal plant the Thunder God Vine, and Minelide, which is water soluble, inhibit the ATPase function of XPB by covalently modifying the cysteine residue at position 342 (C342) [213]. Triptolide triggers global transcriptional inhibition via CDK7-dependent degradation of the RPB1 subunit of RNA Pol II, which may contribute to its capacity to inhibit NER activity and induce apoptosis in lung cancer cells [214]. In vivo experiments showed that the binding of TPL to XPB destabilizes its interaction with the p52 and p8 subunits of the core of TFIIH, causing the degradation of these three subunits that form a submodule without affecting the rest of the TFIIH subunits [215]. Two other molecules, i.e., spironolactone, an antagonist of aldosterone, and ZL-12A, a chemically defined spirocycle acrylamide, also target XPB and covalently bind C342 [216,217]. Unlike triptolide, which induces the degradation of the RPB1 subunit of RNA Pol II, the two molecules promote FBXL18-dependent XPB degradation without causing substantial effects on cancer cell growth, a property that, for spironolactone, correlates with minimal effects on transcription in comparison to triptolide. Interestingly, spironolactone was shown to abrogate the NER capacity of representative bladder cancer cell lines and to increase platinum-induced cytotoxicity in patient-derived organoids, supporting the notion of repurposing spironolactone for improving the chemotherapy response of neoadjuvant chemotherapy in patients with muscle-invasive bladder cancer [218]. Recent drug repurposing screens have also identified new and diverse functions for SP as a simulator of tumor immunosurveillance and as an inhibitor of viral infection [219,220,221].

Inhibiting kinases is a critical strategy in combating cancer because kinases play pivotal roles in regulating cell signaling pathways that control cell growth, division, survival, and differentiation [222]. CDK7’s dual role in transcription and cell cycle regulation makes this kinase an attractive drug target in cancer therapy. Inhibiting CDK7 is broadly cytotoxic, but it is particularly harmful in cells that exhibit transcriptional addiction, relying heavily on continuous oncogene expression and hyperactive transcriptional machinery [223,224]. By targeting CDK7, it was, for instance, possible to selectively target cancers with high oncogene dependence, such as T-cell acute lymphoblastic leukemia (T-ALL) reliant on the oncogenic transcription factor Runt-related transcription factor 1 (RUNX1) [225], MYC-driven cancers, and triple-negative breast cancer [226].

As drug development progresses, numerous CDK7 inhibitors have been developed, primarily targeting the ATP-binding pocket. Compounds such as THZ1 and SY-1365 are typical class I inhibitors that covalently bind to the ATP site of CDK7, downregulating its phosphorylation of RNA pol II [225]. Other CDK7 inhibitors, such as ICEC0942, YKL-5-124, and QS1189, exhibit antitumor activity by interfering directly with cell cycle progression, leading to cell cycle arrest and apoptosis [227]. Additionally, some CDK7 inhibitors, including THZ1, combine effects on both transcription and cell cycle progression. Four CDK7-specific inhibitors—ICEC0942, SY-1365, SY-5609, and LY3405105—are currently in Phase I/II clinical trials.

The discovery of promising anti-cancer drugs targeting XPB and CDK7 has confirmed TFIIH as a viable target for developing the next generation of small-molecule anti-cancer drugs. While XPD, the third catalytic subunit, is often considered as a biomarker in various cancers [228], to date, molecules targeting XPD helicase activity have not been identified.

Protein–protein interactions (PPIs) create complex cellular networks essential for many crucial biological processes, providing a promising avenue for drug discovery. In cancer research, several PPI modulators have entered clinical trials in recent years, and venetoclax, which targets Bcl-2 family proteins, has been approved for treating various types of leukemia [229]. The p8/TTD-A protein is a TFIIH subunit that participates in the control of the steady state level of TFIIH and its DNA repair activity by stabilizing the complex through interactions with p52 and XPB (see Section 8, The Molecular Basis of TFIIH-Associated Diseases). By leveraging the protein–protein interface conserved in the p8/TTD-A homodimer and p8/TTD-A/p52 heterodimer states, a fragment-screening strategy combined with biophysical analysis identified small molecules that bind to the dimerization interface of p8/TTD-A, leading to its destabilization, as confirmed by biophysical and NMR studies [135,230]. Two of these molecules were also shown to reduce the intracellular concentration of TFIIH and its transcriptional activity to levels comparable to those seen in individuals with trichothiodystrophy due to mutations in TTD-A [135].

## 10. Conclusions

Despite significant progress in our understanding of NER and of the pivotal role of TFIIH over the past few decades, much remains to be explored, particularly regarding its dynamics in the cellular context. NER is a highly regulated and rapid process that operates differently within cells, especially in the chromatin context, compared to in vitro systems. Therefore, it is crucial to investigate TFIIH and the factors governing its function during NER in the complex environment of the cell.

Although recent groundbreaking structural and biophysical studies have led to marked progress in the mechanistic characterization of the TFIIH complex in transcription initiation and DNA repair, transient complexes in different functional states are still unexplored. Gaining insights into this structure will reveal important information about the plasticity of the complex and its ability to adapt to various cellular processes, including transcription, telomere replication, mitosis, and mitochondrial genome repair.

Finally, a more detailed exploration of the other pathways in which TFIIH is involved is essential. Such research would provide a deeper understanding of the diverse phenotypes observed in patients with mutations in this complex, potentially guiding new therapeutic strategies.

## Data Availability

No new data were created or analyzed in this study.
